# Prevalence of central sensitisation associated symptoms and associations with treatment outcomes in surgical, interventional and injection-based treatment for patients with chronic spinal pain

**DOI:** 10.1177/20503121251387062

**Published:** 2025-10-18

**Authors:** Mario Giuseppe Zotti, Keenan Janfada-Balov, William Roger Peters, Luke C. Smith, Evelyne Rathbone, Allan Stirling

**Affiliations:** 1 Bond University, Faculty of Health Sciences and Medicine, Gold Coast, Australia; 2 Back/Neck, Gold Coast, QLD, Australia; 3 Gold Coast Hospital and Health Service, Gold Coast, QLD, Australia

**Keywords:** Spine, back pain, surgery, central sensitisation, satisfaction, central sensitisation inventory

## Abstract

**Objectives:**

This study aimed to assess the prevalence of symptoms potentially related to central sensitisation (CS) in patients with spinal pain and explore its association with patient-reported treatment outcomes.

**Methods:**

This study was designed as a single-centre prospective cohort study evaluating 496 patients undergoing surgical and non-surgical management for spinal pain between 2020 and 2023. Patients with symptoms lasting more than three months were assessed for symptoms associated with CS using the validated Central Sensitisation Inventory (CSI) before treatment. Treatment satisfaction was then assessed using a 5-point Likert scale. Complete data on patient demographics were available for 492 patients.

**Results:**

The prevalence of a CSI score of 40+ was 49.9%. Non-surgical patients had a higher median CSI score (42, IQR: 32–49) compared to surgical patients (34.5, IQR: 24–48) (*p* = 0.001). A moderate negative correlation was found between CSI scores and Likert scores (*r* = −0.69, *p* < 0.001). Multiple regression analysis showed that both treatment type and CSI scoring significantly impacted satisfaction scores (*p* < 0.001). Logistic regression revealed that higher CSI scores (40+) decreased treatment satisfaction (OR = 0.09) (*p* < 0.001). Where post-treatment patient-reported outcome scores were available, the cohort of patients with CSI⩾40 compared to the <40 cohort had a lower proportion of patients who achieved minimum clinically important difference and patient acceptable symptom state for both Neck Disability Index and Oswestry Disability Index (*p* < 0.05).

**Conclusions:**

Overall, high CSI scores were common in patients with chronic spinal pain and were significantly associated with treatment dissatisfaction. Higher CSI scores should be considered when selecting treatment and managing patient expectations.

## Introduction

Chronic pain is a complex and multifactorial condition involving biological, psychological and social contributors. One proposed mechanism contributing to persistent pain is central sensitisation (CS), defined by the International Association for the Study of Pain as ‘increased responsiveness of nociceptive neurons in the central nervous system to their normal or subthreshold afferent input’. This mechanism may lead to altered pain processing, resulting in symptoms such as hyperalgesia, which is an amplified response to noxious stimuli, and allodynia, defined as pain from a stimulus that does not normally provoke pain.^1^ However, hyperalgesia is a non-specific clinical feature and can also arise from peripheral sensitisation, thus limiting its diagnostic utility in inferring a central mechanism.^2^

The role of CS in chronic pain remains theoretical and unproven, though it is frequently discussed in the literature as a possible contributor to persistent pain syndromes.^3^ Due to the absence of an objective diagnostic test for CS in clinical practice, symptom-based approaches are used for identifying patients who may exhibit features consistent with central sensitivity syndromes. As a result, CS can be thought to be an underlying mechanism in numerous chronic pain conditions, and thus, the recognition of CS may have benefits in clinical practice. Symptoms potentially related to CS were also found to interact with numerous psychological factors, including anxiety and depression, thereby contributing to the persistence of chronic pain conditions.^4^ Thus, accounting for symptoms potentially related to CS in the management of chronic pain conditions should be considered by clinicians. Further, recognition of CS-related symptoms can have utility in predicting a patient’s response to and appropriateness for treatments.^5^

This single-centre cohort study aimed to investigate the prevalence and predictive value for treatment satisfaction of CS in patients who presented for and subsequently underwent treatment for spinal pain. The patients in this study were managed through differing treatment modalities, including conservative management (corticosteroid injection), pain procedural management and open surgical treatment. The authors present a novel perspective on the predictive benefits of measuring pain phenotypes in a large spinal pain population.

## Methods

### Study design and setting

This prospective cohort study evaluated 496 consecutive patients who presented for treatment for chronic spinal pain (>3 months in duration) to a single surgeon’s elective spinal clinic for either surgical or non-surgical treatment of spinal pain between 2020 and 2023. These patients were referred by general practitioners and other specialists following failure of conservative treatment for their chronic pain. Lifestyle modifications and physical therapy were routinely prescribed, but the details of the full range of these interventions were not comprehensively measured or detailed. Neuromodulating medications were not routinely prescribed by the surgeon; however, a substantial proportion of patients were either already taking these medications or were initiated on them following surgical assessment and recommendation. Specific details regarding these prescriptions were not documented. Recruited patients were treated through the clinic and gave written informed consent for the use of their data and outcome scorings in future research, per the ethics approval. Patients were allocated to a treatment group based on the spinal surgeon’s routine clinical practice following a full history and clinical examination. Conservative and pain interventional treatments are routinely offered before surgical intervention is entertained, outside of emergency surgical indications. The spinal surgeon was blinded to the Central Sensitisation Inventory (CSI) score at the time of treatment planning.^6^ Patients were excluded if they did not provide consent or if no follow-up review was completed. Demographics, including age, sex and socio-economic status (SES), were assessed at baseline, with the Charlson Comorbidity Index (CCI) being utilised to assess for the presence of comorbidities.^7^ SES was classified according to the Australian Bureau of Statistics’ Socio-Economic Indexes for Areas (SEIFA) framework, based on demographic information provided to the clinic. This included listed occupation (used to estimate median income), employment status (pensioner vs. self-funded retiree), level of private health insurance coverage, and residential postcode. Using these indicators, participants were stratified into three SES categories: low: annual income under AUD $48,000 or holding a Pension/HCC card; middle: annual income between AUD $48,000 and $180,000 or self-funded retiree; and high: characterised by a combination of high-level private insurance, residence in a high-SEIFA postcode, and employment in an executive or business ownership role. Ethics approval was gained for research into variables affecting recovery and treatment outcomes in patients with spinal pain through the Bond University Human Research Ethics Committee with reference MZ00044.

### Measurement properties and outcomes

Central sensitisation-related symptoms were assessed pre-treatment using the validated 100-point CSI patient-reported outcome tool. The CSI is not a diagnostic tool for CS, but rather a validated questionnaire designed to identify symptom clusters associated with central sensitivity syndromes. The CSI assesses the extent of 25 emotional and somatic symptoms, scoring each item from 0 (never) to 4 (always) to produce a score out of 100.^6^ CSI scores were graded using validated severity levels as subclinical (0–29), mild (30–39), moderate (40–49), severe (50–59) and extreme (60–100). According to Neblett and colleagues, a score of 40 or higher on the CSI has been proposed as a reasonable cut-off to alert clinicians that a patient’s symptoms may indicate the presence of CS.^8,9^ Patient-reported outcome scores (PROMS), being the Oswestry Disability Index (ODI) and Neck Disability Index (NDI), were utilised pre-treatment to assess functional disability in patients with lower back pain and neck pain, respectively.^10,11^ The primary outcome assessed in this study was treatment satisfaction, which was assessed using a 5-point modified Likert scale. Treatment satisfaction was assessed as per treatment-specific follow-up protocols; at 6–8 weeks for those who underwent non-surgical and pain procedural management and at 2–3 months for open surgical management. This was recorded by the patient following surgeon review by the clinic staff, either in writing, if for an in-person review, or recorded in a subsequent phone call by clinic staff, for telephone conducted reviews, using the question ‘How satisfied were you with the outcome of your treatment’ explaining the five different modified Likert options verbally. A score of five equated to ‘very satisfied’, four equal to ‘satisfied’, three was impartial, two was ‘dissatisfied’ and one was ‘very dissatisfied’. Where complete pre- and post-treatment PROMs were available for analysis, a Minimum Clinically Important Difference (MCID) for ODI of 14 and for NDI of 30% change was applied. For Patient Acceptable Symptom State (PASS), final scores of <25 for ODI and <21 for NDI were applied, consistent with the published literature.^12–19^

### Statistical analysis

Descriptive statistics were presented as counts and percentages for categorical variables, and mean (SD) or median (IQR) for continuous variables, depending on data distribution. Normality was assessed using a combination of histograms, normal Q-Q plots and the Shapiro–Wilk test. Differences between groups were assessed using a Chi-Squared test for categorical variables and the independent *t*-test for normally distributed variables or Mann–Whitney *U* test for skewed continuous variables. Differences in normally distributed variables between multiple groups were assessed using a one-way ANOVA, followed by post-hoc tests to determine any significant pairwise differences. Correlations between variables were evaluated using Spearman rank order correlation. Multiple linear regression analysis was performed to identify factors contributing to patient-reported treatment outcomes with the modified Likert score as the dependent variable. Diagnostic checks were carried out, including collinearity and goodness-of-fit. For analysis, patients were grouped as surgical versus non-surgical, as this represents the main clinical decision point and preserved statistical power. Patients were also recategorised based on CSI scores using the validated cut-off score of 40, with those ⩾40 being classed as having CS for the purpose of the logistic regression. Statistical significance was set at *p* < 0.05 without multiple comparison correction due to the exploratory nature of the analyses performed in this study. Statistical analysis was performed using Jamovi™ ver. 2.2.5.0.6.

## Results

### Patient demographics

Patient demographics are presented in Table 1. Overall, complete demographic data was available for 492 patients who completed pre- and post-intervention scoring. Among these patients, 190 underwent open surgical treatment, including spinal decompression and both single and multiple level reconstruction, including fusion and/or disc replacement. A further 146 patients underwent pain procedural treatment, such as radiofrequency treatments (e.g. facet denervation, dorsal root ganglion pulsing, dorsal column stimulation) for their spinal pain, with the remaining 160 patients being managed conservatively with corticosteroid injections, being deemed the upper limit for conservative treatment. The median age was similar across the treatment groups, with the median age of the surgical group being 60 years versus 57.5 years for the non-surgical group. There was a statistically significant difference in the sex distribution among the treatment groups ( $\chi_{1}^{2}$ = 11.4, *p* < 0.001) with the surgical group comprising a higher proportion of males of 69.0% compared to females at 31.0%. In comparison, the non-surgical group comprised 53.4% males and 46.6% females. SES showed no statistically significant difference among the treatment groups ( $\chi_{3}^{2}$ = 4.93, *p* = 0.18) with a similar distribution across the SES categories. There was a statistically significant difference between the treatment groups in the distribution of the CCI ( $\chi_{2}^{2}\;=\;$ 6.74, *p* = 0.03) as a higher proportion of patients with an index >9 (3.7% versus 0.7%) was in the surgical group. 32 of the 492 patients were evaluated and treated for both cervical and lumbar symptoms (6.5%).

**Table 1.** Patient demographics.

	Total (*N* = 492)	Non-surgical (*n* = 305)	Surgical (*n* = 187)	*p*-value
Age (years), median (IQR)	58.0 (44.0-69.0)	57.5 (44.0-68.0)	60.0 (44.3-70.0)	0.50^a^
Sex				< 0.001*^b^
Female	200 (40.7%)	142 (46.6%)	58 (31.0%)	
Male	292 (59.3%)	163 (53.4%)	129 (69.0%)	
SES				0.18^b^
Compensable	153 (31.1%)	101 (33.1%)	52 (27.8%)	
Low	155 (31.5%)	100 (32.8%)	55 (29.4%)	
Mid	151 (30.7%)	87 (28.5%)	64 (34.2%)	
Highest	33 (6.7%)	17 (5.6%)	16 (8.6%)	
CCI				0.03* ^b^
<5	418 (85.0%)	265 (86.9%)	153 (81.8%)	
5–9	65 (13.2%)	38 (12.5%)	27 (14.4%)	
>9	9 (1.8%)	2 (0.7%)	7 (3.7%)	

CCI = Charlson Comorbidity Index; SES = Socio-economic Status.

a Mann–Whitney *U* statistic: 28019; effect size: 0.04 (very small).

b Chi-square test.

* Statistically significant *p* < 0.05.

### CSI scores

A summary of the distribution of CSI scores between treatment groups is presented in Table 2. The median CSI score across all the patients was 39.5, with scores ranging from 8 to 63. Patients in the non-surgical management group had a higher median CSI score of 42 (IQR: 32–49) in comparison to the surgical group with a median CSI score of 34.5 (IQR: 24–48), with this difference being statistically significant (*p* = 0.001), although the clinical significance of this difference is unclear. In terms of distribution, there was a statistically significant association between CSI categories and treatment group ( $\chi_{4}^{2}$ = 27.3, *p* < 0.001). Among the CSI score severity levels, a higher proportion of those managed surgically fall into the subclinical CSI score range (CSI 0–29) at 37.9% compared to 18.6% for non-surgical patients. Further, a higher proportion of non-surgical patients were classified as having moderate (CSI 40–49) and severe (50–59) CSI scores at 35% and 19% respectively. In contrast, 20% of surgical patients had moderate CSI scores, and 17.4% had severe scores. The proportion of patients with extreme (CSI 60+) scores was similar in both treatment groups at 2% for non-surgical patients and 3.2% for surgical patients. CSI scoring was not repeated post-treatment.

**Table 2.** Pre-treatment CSI, ODI and NDI scores by treatment group.

	Total (*N* = 496)	Non-surgical (*n* = 306)	Surgical (*n* = 190)	*p*-value
CSI score (0–100)				0.001*^a^
Median (IQR)	39.5 (29.0–48.3)	42.0 (32.0–49.0)	34.5 (24.0–48.0)	
Range	8–63	8–61	9–63	
CSI categories				<0.001*^b^
Subclinical (0–29)	129 (26.0%)	57 (18.6%)	72 (37.9%)	
Mild (30–39)	119 (24.0%)	78 (25.5%)	41 (21.6%)	
Moderate (40–49)	145 (29.2%)	107 (35.0%)	38 (20.0%)	
Severe (50–59)	91 (18.3%)	58 (19.0%)	33 (17.4%)	
Extreme (60+)	12 (2.4%)	6 (2.0%)	6 (3.2%)	
	Total (*N* = 401)	Non-surgical (*N* = 243)	Surgical (*N* = 158)	*p*-value
ODI				< 0.001*^c^
Mean (SD)	36.9 (8.7)	35.4 (9.4)	39.3 (6.9)	
Range	4–76	4–76	16–62	
	Total (*N* = 123)	Non-surgical (*N* = 77)	Surgical (*N* = 46)	*p*-value
NDI				0.27^d^
Mean (SD)	38.1 (7.9)	37.5 (8.6)	39.2 (6.6)	
Range	22–68	22–68	24–54	

CSI = Central Sensitisation Inventory; ODI = Oswestry Disability Index; NDI = Neck Disability Index.

a Mann–Whitney *U* statistic: 24,051; effect size: −0.17 (very small).

b Chi-square test.

c *t*-statistic: −4.8; effect size: −0.48 (small).

d *t*-statistic: −1.11; effect size: −0.21 (small).

* Statistically significant *p* < 0.05.

Overall, females had a higher median CSI at 46 (IQR: 36.8-51.0) compared to males at 34 (IQR: 24–45). Females also had a lower median Likert score at 3 (IQR: 2–4) compared with males at 4 (IQR: 3–4). Although both differences were statistically significant (*p* < 0.001), the effect size was small (CSI: −0.43; Likert: 0.27).

### ODI and NDI scores

**Figure 1.**
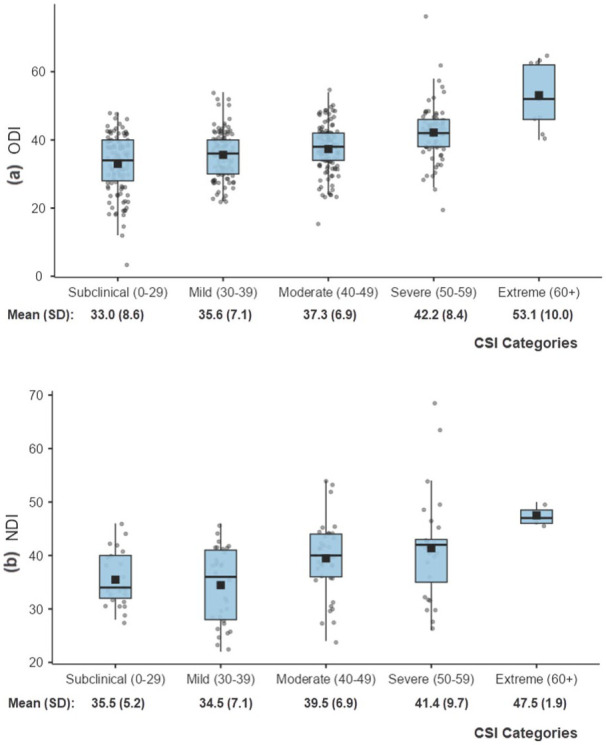
ODI (Oswestry Disability Index) and NDI (Neck Disability Index) scores distribution by CSI (Central Sensitisation Inventory) categories; the mean is represented by a solid square. (a) ODI: All pairwise differences were statistically significant except for the pairs: subclinical versus mild, mild versus moderate and severe versus extreme. (b) NDI: All pairwise differences were statistically significant except for the pairs: subclinical versus mild, subclinical versus moderate, moderate versus severe, moderate versus extreme and severe versus extreme.

The distribution of pre-treatment ODI and NDI scores based on treatment group is summarised in Table 2. Overall, the mean ODI score reported in surgical patients was higher at 39.3 (SD = 6.9) compared to non-surgical patients at 35.4 (SD = 9.4), with this difference being statistically significant (*p* < 0.001). There was no statistically significant difference in the mean NDI scores between the surgical and non-surgical groups. Figure 1(a) and (b) display the distribution of ODI and NDI scores, respectively, based on the CSI severity category. As CSI score severity increased, mean ODI scores also increased (*F* [4, 54.3] = 18.6, *p* < 0.001). Patients in the subclinical range had the lowest mean ODI score of 33.0 (SD = 8.6), and patients in the extreme category had a significantly higher mean score of 53.1 (SD = 10.0, *p* = 0.002). A similar trend of increased disability with increased CSI score severity is also observed in the NDI scores (*F* [4, 118] = 5.99, *p* < 0.001). The lowest mean NDI score was in those with a mild CSI score at 34.5 (SD = 7.1), with the mean NDI score being significantly higher in those with an extreme CSI score at 47.5 (SD = 1.9, *p* = 0.009).

Among the 492 patients with completed baseline CSIs, 32 patients underwent both cervical and lumbar treatment. For analysis purposes, we considered each PROM (NDI for cervical, ODI for lumbar) only once per patient and did not duplicate any individual’s data. Thus, while the theoretical maximum number of outcome entries was 524, each score was linked to a distinct anatomical region and PROM. We treated ODI and NDI scores as separate outcome variables and analysed them independently, without combining or duplicating patient-level data across outcomes.

For the patients with lumbar symptoms, the proportion of each CSI severity group post-treatment reaching PASS for ODI was as follows: Subclinical 76%, Mild 53%, Moderate 44%, Severe 37% and extreme 11%. Comparing the group of patients with CSI <40 group (*n* = 143) to ⩾40 group (*n* = 193), there was a statistically significant difference in proportion of the group who did not reach MCID, with 46/143 (31.2%) of CSI <40 compared to 127/193 (65.8%) in the CSI⩾ 40 groups, respectively (*X*^2^, *p* < 0.05).

For the patients with cervical symptoms, regarding the proportions of each CSI severity group achieving MCID, these were Subclinical 85.0%, Mild 63.3%, Moderate 51.4%, Severe 14.3%, Extreme 0%, respectively. When comparing the CSI <40 group (*n* = 50) to the CSI⩾40 group (*n* = 57), there was a significant difference with 14/50 (28.0%) did not achieve PASS in low CSI group (<40) while 44/57 (77.2%) did not achieve PASS in the high CSI group (⩾40) (*X*^2^, *p* <0.05).

### Correlations

Table 3 demonstrates the correlation between CSI scores and the other variables included in this study. Overall, there are significantly low positive correlations between CSI scores and pre-treatment ODI scores (*r*_s_ = 0.40, *p* < 0.001) and NDI scores (*r*_s_ = 0.37, *p* < 0.001), suggesting an association between CSI scores and higher levels of functional disability pre-treatment. Further, there was a moderate negative correlation between CSI and post-treatment Likert scores (*r*_s_ = −0.69, *p* < 0.001), indicating that higher CSI scores are associated with lower treatment satisfaction. A low, significantly negative correlation between CSI scores and SES is also demonstrated (*r*_s_ = −0.22, *p* < 0.001). There are also significantly low negative correlations between Likert scores and ODI scores (*r*_s_ = −0.23, *p* < 0.001) and NDI scores (*r*_s_ = −0.30, *p* < 0.001). Correlations were not performed on the post-treatment PROMs data due to the incomplete data set.

**Table 3.** Spearman rank correlation matrix.

	CSI score	ODI	NDI	Likert	SES
ODI	0.40**	—			
NDI	0.37**	0.36	—		
Likert	−0.69**	−0.23**	−0.30**	—	
statuses	−0.22**	0.02	−0.004	0.25**	—
CCI	0.07	0.20**	0.16	−0.05	0.13*

CSI = Central Sensitisation Inventory; CCI = Charlson Comorbidity Index; SES = socio-economic status; ODI = Oswestry Disability Index; NDI = Neck Disability Index.

* *p* < 0.05. ** *p* < 0.001.

### Multiple linear regression results

Multiple linear regression was performed to identify the factors influencing post-treatment satisfaction and is depicted in Table 4. The multiple linear regression model was relatively strong (moderate *R*^2^ value of 0.53), explaining 52.9% of the variance in the Likert score as the outcome. The results of the regression model demonstrated that CSI scores were significantly associated with the Likert scores. After accounting for all other factors in the model, all CSI score severity categories yielded lower satisfaction, compared with Subclinical, and the differences were statistically significant (*p* < 0.001). Patients with mild and moderate CSI scores demonstrated significant decreases in post-treatment Likert scores, with a 0.49 point decrease (*p* < 0.001, 95%CI [−0.67, −0.32]) for the mild category and a 1.00 point decrease (*p* < 0.001, 95%CI [−1.18, −0.83]) in the Likert scores for those in the moderate category, in relation to the Subclinical group. Those categorised as having severe and extreme CSI scores were also associated with significant decreases in Likert scores, with a 1.56 point (*p* < 0.001, 95%CI [−1.75, −1.37]) and 1.99 point (*p* < 0.001, 95%CI [−2.40, −1.59]) decrease in Likert scores, respectively, when compared with the Subclinical group. Overall, the results of this multiple linear regression model demonstrate that increased CSI score is significantly associated with a decrease in post-treatment satisfaction, in the presence of all the other factors entered in the model.

**Table 4.** Multiple Linear Regression results depicting associations between variables, with the Likert score as the dependent variable.

			95% confidence interval				
Predictor	Estimate	Standard error	Lower	Upper	*t*	*p*-value	Stand. estimate
Intercept^a^	3.75	0.14	3.45	4.02	27.1	< .001*	
Treatment group							
Surgical (vs Non-surgical)	0.56	0.07	0.43	0.69	8.6	< .001*	0.58
Age (years)	0.003	0.002	−0.001	0.008	1.3	0.18	0.05
SES (Reference: compensable)							
Low	0.09	0.08	−0.07	0.25	1.1	0.28	0.09
Mid	0.16	0.08	−0.009	0.32	1.9	0.06	0.17
Highest	0.19	0.14	−0.07	0.46	1.4	0.16	0.20
CCI (Reference: <5)							
5–9	−0.06	0.10	−0.26	0.13	−0.6	0.53	−0.07
>9	−0.68	0.23	−1.14	−0.22	−2.9	0.004*	−0.70
CSI Categories (Reference: Subclinical (0–29))							
Mild (30–39)	−0.49	0.09	−0.67	−0.32	−5.5	< .001*	−0.51
Moderate (40–49)	−1.00	0.09	−1.18	−0.83	−11.3	< .001*	−1.04
Severe (50–59)	−1.56	0.10	−1.75	−1.37	−16.0	< .001*	−1.61
Extreme (60+)	−1.99	0.21	−2.40	−1.59	−9.6	< .001*	−2.06

CSI = Central Sensitisation Inventory; CCI = Charlson Comorbidity Index; SES = Socio-economic Status. Model fit measures: *R*^2^ = 0.53, *F* (10, 476) = 52.1, *p* < 0.001.

a Represents reference level.

* Statistically significant *p* < 0.05.

## Discussion

This study investigated the prevalence of CS-related symptoms among patients with chronic spinal pain and examined their association with patient-reported outcomes following surgical and non-surgical treatment. The findings reveal a high prevalence of clinically significant CS-related symptoms, with 49.9% of patients scoring ⩾40 on the CSI. Importantly, higher CSI scores were associated with reduced treatment satisfaction and increased functional disability, highlighting the potential role of CS-related symptoms in influencing treatment outcomes and informing clinical decision-making.

Compared to previous studies evaluating spinal surgery populations, this cohort demonstrated a markedly higher prevalence of CS. A previous study reported a CS prevalence of 13.2% with a mean CSI of 23.6 (SD = 13.5), substantially lower than the present cohort’s median of 34.5 (IQR:24–48).^20^ This discrepancy may be attributed to differences in study population characteristics. Our sample consisted of patients referred for specialist intervention after failed conservative management, likely enriching for individuals with complex and chronic pain presentations. This referral bias may explain the elevated CSI scores, especially in the non-surgical group, despite clinician blinding to CSI at the time of treatment allocation.

This study demonstrated a moderate negative correlation between CSI scores and post-treatment satisfaction (*r_s_* = −0.69, *p* < 0.001), with higher CSI severity strongly predictive of dissatisfaction (Table 3). Patients with extreme CSI scores experienced the lowest satisfaction (median Likert = 2), and only 11% achieved PASS in ODI outcomes, underscoring the clinical impact of CS on perceived treatment efficacy. These findings align with the literature across musculoskeletal conditions. In total knee arthroplasty, higher CSI scores predict persistent postoperative pain and dissatisfaction.^21,22^ Similar trends are seen in shoulder and hip pain populations, where CS-related symptoms impair recovery despite surgical or conservative interventions.^23–26^ Other studies looking at physical therapy for the management of chronic back pain and knee osteoarthritis also demonstrated a higher prevalence of clinically important CS-related symptoms in those who do not respond to treatment and decreased quality of life.^27^ These studies demonstrate that the presence of CS-related symptoms in patients is associated with decreased satisfaction following both surgical and non-surgical treatment. Analysis in this study of post-treatment NDI and ODI scores was limited by missing data among the cohort, but despite this, it is evident that patients with higher CSI (⩾40) were significantly less likely to achieve MCID and PASS compared to those with lower CSI (<40). Only 37.0% and 11.4% of patients in the severe and extreme categories reached ODI MCID, and only 14.3% and 0% reached NDI MCID, respectively.

A notable addition in this analysis is the exploration of SES in relation to CS-related symptoms and outcomes. We observed a small but statistically significant negative correlation between SES and CSI scores (*r_s_* = −0.22, *p* < 0.001), suggesting that patients from lower SES backgrounds may be more likely to exhibit symptoms consistent with CS syndromes. Lower SES is a recognised social determinant of health that influences pain chronicity, psychological distress, and access to multidisciplinary care.^4,21^ Despite this correlation, SES was not a statistically significant factor in predicting satisfaction within the multivariable linear regression model. However, in the logistic regression model = patients in the mid-SES category (vs compensable) were more than twice as likely to be satisfied (OR = 2.20, *p* = 0.012), suggesting a possible nuanced role of socio-economic context in shaping treatment experience and perceived benefit. Future studies should explore this interaction further, potentially using more granular SES measures and qualitative patient perspectives.

Notable sex-based differences were observed in the distribution of CSI score severity. In this study, females had significantly higher mean CSI scores than males, a trend consistent with findings in previous literature.^28^ For example, a study examining preoperative CS-related symptoms and surgical outcomes in patients with lumbar spinal stenosis reported a much lower overall prevalence of CS at 7.3%, with a mean CSI score of 21.8 (SD = 11.3).^28^ Further, they found higher mean CSI scores in females compared to males. The substantially higher CS-related symptom prevalence observed in the current cohort may be attributed to several factors, including the presence of comorbid psychological conditions and demographic differences.^29^ Specifically, the higher proportion of female patients in this study may have contributed to the elevated CSI scores reported. In addition, observed differences in sex distribution between treatment groups, with a significantly higher proportion of males in the surgical group, may reflect underlying clinical referral patterns and decision-making processes. The reasons for differences in presentation and severity of CS-related symptoms between genders remain unclear, though occupational and lifestyle factors may play a role. Existing literature supports this trend, showing a significant positive correlation between CSI scores and female sex.^30^ The definitive impact of sex differences on CS requires further investigation.

Furthermore, functional rehabilitation, including exercise and psychosocial therapy, is frequently recommended for chronic pain, with CSI often used to monitor treatment efficacy.^31^ One study found significant associations between previous CS syndromes and rehabilitation outcomes in a spinal pain population, with improvements in CSI scores following discharge from functional rehabilitation.^31^ This suggests that preoperative interventions targeting CS could improve outcomes for surgical patients. Although this study found higher satisfaction levels in surgically managed patients, the comparative effectiveness of surgical versus non-surgical treatments for patients with CS remains unclear. Further research is needed to explore this area and determine how best to tailor treatment approaches for individuals with CS to optimise patient satisfaction and functional recovery.

In addition to negatively impacting patient-reported treatment outcomes, it was also demonstrated that significant CSI scoring was correlated with higher levels of functional disability pre-treatment, as assessed by the NDI and ODI. This finding is supported by previous studies, with it being found that the presence of CS-related symptoms is associated with higher levels of functional disability and decreased quality of life.^32,33^ This increased functional disability mainly has psychosocial implications, with CS-related symptoms and high CSI scores, therefore, being associated with an increased incidence of anxiety, depression and pain catastrophising.^34,35^ It could also reflect the biopsychosocial phenotype of the patient and their reaction to painful stimuli as being interrelated. This reinforces the importance of early identification of likely CS symptomatology and management of pain conditions that are associated with CS. In terms of functional disability, further research is required to determine whether management of musculoskeletal pain conditions leads to decreased levels of CS and whether this leads to a subsequent improvement in functional disability. In addition, the optimal management option for patients with high CS has not been demonstrated and validated in the literature. Therefore, there is a need for consensus on the optimal management of patients with high CS.

This study has several limitations. First, CSI was assessed only pre-treatment, preventing evaluation of post-treatment changes. Second, the lack of data on psychological variables such as depression, anxiety and catastrophising limits the interpretation of CS-related distress.^34,35^ Given their established correlation with CSI, future studies should include these measures to better characterise the biopsychosocial phenotype. Grouping treatment types into broad surgical versus non-surgical categories introduced heterogeneity, and more granular analysis by procedure type is warranted. In addition, patient selection into treatment pathways reflects real-world clinical judgment, but may introduce unmeasured confounding, especially given the observed differences in CSI scores, sex and SES between groups. Although three treatment pathways were represented, non-surgical treatments (injections and interventional procedures) were combined into a single group, which may obscure potential subgroup differences. While CSI scores were not used to guide treatment decisions and the clinician was blinded to them, patients exhibiting more diffuse, non-structural pain patterns, reflecting potential CS, may have been less likely to be offered surgical intervention. This reflects real-world clinical practice and may have contributed to the CSI score difference between groups. Further, this study did not perform a formal sample size calculation. Although the sample was large and prospectively collected, the lack of a prior power analysis may limit interpretation of subgroup findings and increase the risk of type II error in underpowered comparisons.

In addition, outcome completeness was limited. While post-treatment satisfaction was consistently assessed using a 5-point Likert scale, validated functional outcome measures (ODI and NDI) were incompletely captured due to missing data. This was largely attributable to patients who opted for telephone follow-up, where PROMs were not routinely completed. Consequently, reliance on a single-item satisfaction measure limited the ability to comprehensively evaluate functional outcomes. A complete set of PROMs would have strengthened the analysis by allowing more standardised statistical comparisons and enhanced external validity. Future studies should aim for full PROMs collection to better assess the impact of CSI on treatment outcomes and to facilitate cross-study comparisons.

Lastly, the study design did not account for procedural nuances or previous treatment history, and surgeon selection of treatment pathways, although reflective of typical practice, was subject to individual clinical judgment. These factors limit generalisability and may obscure how CS interacts with different stages or types of intervention. The study design did not account for specific nuances within the type of surgical treatment or whether the patient had previously had failed conservative, procedural treatment, but instead looked only at the metric of ‘surgery’ and the final outcome, thus missing an opportunity to analyse the different directions function and sensitisation may take at different points in the patient journey. Replication of this study may yield different patterns of CS-related symptom prevalence across treatment groups, reflecting variations in clinician decision-making and the growing consideration of CS in treatment planning.

These findings reinforce the value of incorporating CS screening into pre-treatment assessments. Identifying patients with elevated CSI scores may enable clinicians to provide better expectation management, allocate appropriate treatment pathways and potentially introduce targeted interventions such as pain neuroscience education, cognitive behavioural therapy, or multimodal rehabilitation programmes.^31^ While high satisfaction can still be achieved in some patients with significant CS, the proportion is markedly lower, suggesting that these patients may benefit from additional support and individualised care plans. Moreover, the study supports the need for consensus regarding optimal management strategies for patients with CS. Conversely, patients with lower CSI scores responded better across all treatment types, suggesting that CS status may function as a moderating variable.

## Conclusion

This study demonstrates that clinically significant CSI scores are highly prevalent in a tertiary spinal pain cohort and are independently associated with poorer satisfaction and functional outcomes following treatment. Elevated CSI scores were more common in females and those with lower SES, further supporting the biopsychosocial model of chronic pain. Significant CSI scores also demonstrated a positive correlation with worse functional disability prior to treatment. Furthermore, cohorts of patients with high CSI scores have a higher proportion of failure to achieve MCID and PASS post-treatment for ODI and NDI following spinal treatments, respectively. Assessment of potentially CS associated symptoms should be incorporated into clinical workflows to guide treatment planning, patient counselling and resource allocation. Further research is warranted to explore optimal management strategies for patients with high CSI scores and to evaluate whether interventions targeting CS can improve long-term outcomes.

## Supplemental Material

sj-pdf-1-smo-10.1177_20503121251387062 – Supplemental material for Prevalence of central sensitisation associated symptoms and associations with treatment outcomes in surgical, interventional and injection-based treatment for patients with chronic spinal pain Supplemental material, sj-pdf-1-smo-10.1177_20503121251387062 for Prevalence of central sensitisation associated symptoms and associations with treatment outcomes in surgical, interventional and injection-based treatment for patients with chronic spinal pain by Mario Giuseppe Zotti, Keenan Janfada-Balov, William Roger Peters, Luke C. Smith, Evelyne Rathbone and Allan Stirling in SAGE Open Medicine

sj-pdf-2-smo-10.1177_20503121251387062 – Supplemental material for Prevalence of central sensitisation associated symptoms and associations with treatment outcomes in surgical, interventional and injection-based treatment for patients with chronic spinal pain Supplemental material, sj-pdf-2-smo-10.1177_20503121251387062 for Prevalence of central sensitisation associated symptoms and associations with treatment outcomes in surgical, interventional and injection-based treatment for patients with chronic spinal pain by Mario Giuseppe Zotti, Keenan Janfada-Balov, William Roger Peters, Luke C. Smith, Evelyne Rathbone and Allan Stirling in SAGE Open Medicine
