# Supplementary material for: Prevalence of central sensitisation associated symptoms and associations with treatment outcomes in surgical, interventional and injection-based treatment for patients with chronic spinal pain
Source: SAGE Open Med. 2025 Oct 18;13:20503121251387062. doi: 10.1177/20503121251387062 (PMC12547141; doi:10.1177/20503121251387062)
Supplement: sj-pdf-2-smo-10.1177_20503121251387062 – Supplemental material for Prevalence of central sensitisation associated symptoms and associations with treatment outcomes in surgical, interventional and injection-based treatment for patients with chronic spinal pain [file sj-pdf-2-smo-10.1177_20503121251387062.pdf]

Modified 5 point Likert outcome score

When considering the outcome of your treatment, please rate your satisfaction below:

1	2	3	4	5
Very dissatisfied	Dissatisfied	Neither Satisfied or dissatisfied	Satisfied	Very Satisfied
